# Relationship between the loss of neutralizing antibody binding and fusion activity of the F protein of human respiratory syncytial virus

**DOI:** 10.1186/1743-422X-4-71

**Published:** 2007-07-10

**Authors:** Changbao Liu, Nicole D Day, Patrick J Branigan, Lester L Gutshall, Robert T Sarisky, Alfred M Del Vecchio

**Affiliations:** 1 Centocor R&D, Inc., 145 King of Prussia Road, Radnor, Pennsylvania, 19087, USA

## Abstract

To elucidate the relationship between resistance to HRSV neutralizing antibodies directed against the F protein and the fusion activity of the F protein, a recombinant approach was used to generate a panel of mutations in the major antigenic sites of the F protein. These mutant proteins were assayed for neutralizing mAb binding (ch101F, palivizumab, and MAb19), level of expression, post-translational processing, cell surface expression, and fusion activity. Functional analysis of the fusion activity of the panel of mutations revealed that the fusion activity of the F protein is tolerant to multiple changes in the site II and IV/V/VI region in contrast with the somewhat limited spectrum of changes in the F protein identified from the isolation of HRSV neutralizing antibody virus escape mutants. This finding suggests that aspects other than fusion activity may limit the spectrum of changes tolerated within the F protein that are selected for by neutralizing antibodies.

## Findings

Human respiratory syncytial virus (HRSV) is the most common cause of serious lower respiratory tract infections in infants and young children worldwide [1]. The F protein represents the major protective antigen conserved between subgroups A and B to which neutralizing antibodies are directed [2-5]. As no vaccines against HRSV are approved, antibodybased prophylaxis with the anti-HRSV F protein antibody palivizumab is the only approved prevention for serious infections in at-risk infants [6,7]. Although resistance to palivizumab currently is not an issue in the clinic [8], wider use of palivizumab may increase this potential. An affinity matured version of palivizumab (motavizumab) is currently in clinical development [9]. Since it is derived from palivizumab, it recognizes a similar epitope [10] thus viral resistance patterns are anticipated to be similar. Palivizumab antibody escape mutants have been studied in vitro and in vivo [11-14]. One of the palivizumab escape mutants (MP4) appears to be more fit in both in vitro and in vivo competitive replication [11], although the reason for this increased fitness is unknown. MAb19 is another murine HRSV neutralizing mAb previously in clinical development [15-17]. Replacement of arginine 429 with serine within antigenic site IV/V/VI confers resistance to MAb19 [15,18]. This antigenic site contains overlapping epitopes as defined by several mAbs [19]. Ch101F is a potent neutralizing antibody generated by grafting the variable regions from murine mAb (101F) onto human IgG1 constant frameworks. By several methods, K433 in antigenic site IV/V/VI was identified as critical for binding [20]. Although mutation of K433 to several residues in recombinantly expressed F protein prevented ch101F binding, only one change (K433T) was identified by mapping of ch101F escape mutant viruses. The same escape mutation was reported for mAb R7.936/4 [19]. To better understand the relationship between resistance to antiHRSV F protein antibodies and F protein function, we used a recombinant approach to generate a panel of mutations in antigenic sites II and IV/V/VI [18] of the F protein and characterized these mutations with respect to expression, neutralizing mAb binding, and fusion activity as previously described [21] in an attempt to better understand the mechanism of action of HRSV neutralizing antibodies and the impact of resistance to these antibodies upon the fusion activity of the F protein. A summary of these results is presented in Table 1. The HRSV neutralizing mAbs palivizumab, MAb19, and ch101F were selected for study as these are potent and are either marketed (palivizumab, Synagis^®^; reviewed in [22]), have been in clinical development (MAb19, RHZ19)[16,17,23], or are good candidates for clinical development (ch101F)[20], respectively. They also recognize one of the two major antigenic sites (site II or site IV/V/VI) within the F protein, and residues in their epitopes critical for binding have been somewhat characterized.

**Table 1:** Summary of results for HRSV F mutations.

**Mutation**	**Processing**	**Percent binding**			**Fusion activity **
		**ch101F**	**palivizumab**	**mAb19**	

Wild-type	Complete	99.9	100.0	99.9	1.0 ± 0.0
K272M	Complete	126.6	3.8	100.5	2.3 ± 0.7
K272N	Complete	95.5	15.3	62.3	2.2 ± 0.5
K272Q	Complete	85.3	30.1	88.7	2.6 ± 0.1
K272T	Complete	70.4	9.1	68.0	1.0 ± 0.1
S275F	Complete	29.4	10.9	31.7	1.6 ± 0.2
T400A	Complete	155.2	126.6	161.2	2.9 ± 0.5
C422S	Complete	159.6	177.5	193.9	1.6 ± 0.6
N428D	Complete	116.2	139.4	121.3	0.75 ± 0.02
N428Q	Complete	117.2	190.2	193.8	1.5 ± 0.02
R429K	Complete	44.6	88.8	11.2	4.0 ± 0.2
R429S	Complete	72.5	150.0	3.9	1.1 ± 0.1
G430A	Complete	79.7	166.5	0.15	1.2 ± 0.2
I431A	Complete	61.9	108.2	81.8	1.6 ± 0.3
I431L	Complete	78.5	80.9	65.5	1.8 ± 0.1
I432L	Complete	74.9	68.9	64.4	1.5 ± 0.2
I432Q	Complete	49.3	60.8	57.9	0.7 ± 0.07
I432T	Complete	191.7	206.9	169.8	1.4 ± 0.3
K433D	Complete	1.1	29.9	1.6	0.4 ± 0.01
K433L	Complete	3.8	88.1	24.5	0.5 ± 0.1
K433N	Complete	2.9	80.7	28.7	2.2 ± 0.5
K433Q	Complete	2.7	60.7	47.4	1.0 ± 0.8
K433R	Complete	-0.6	15.8	23.1	2.0 ± 0.6
K433T	Complete	3.9	64.5	64.4	0.6 ± 0.04
K433S	Complete	69.5	86.3	111.8	0.98 ± 0.19

Processing is defined as relative amounts of F0, F1, and F2, and is described as being equivalent to wild-type HRSV F protein (complete) or reduced. Reactivity with neutralizing mAbs (palivizumab, Mab19, and ch101F) as determined by flow cytometry is reported as percent relative to wild-type HRSV F protein. Cell fusion activity (luciferase activity) is reported relative to wild-type as described in [30]. All values are expressed as relative to wild-type.

Mutations of residues K272 and S275 (K272M, K272N, K272Q, K272T, and S275F) reduced palivizumab binding as expected based upon previous studies [11-14,24], yet retained binding by MAb19 and ch101F confirming that MAb19 and ch101F recognize a different epitope (site IV/V/VI) than palivizumab (site II). These mutations had fusion activity similar to or greater than WT. It is tempting to speculate that this may partially explain the observed increase in replicative fitness of the palivizumab escape mutant MP4 (change of K272 to M) [11] as this mutation had increased fusion activity (2.3 fold relative to WT).

Mutation of R429 to S reduced MAb19 binding (3.9%) as expected based upon previous studies [15,18], yet retained binding by palivizumab confirming that MAb19 and palivizumab recognize different epitopes. Mutation of R429 to K similarly reduced MAb19 binding (11.2%). Binding of ch101F to R429S was somewhat reduced (44.6%), while binding of ch101F to R429K was largely unaffected (72.5% relative to WT) suggesting that these two mAbs recognize somewhat distinct epitopes. Mutation of R429 to S had no effect upon fusion activity. However, interestingly, the structurally conservative mutation of R429 to K caused a 4 fold increase in the fusion activity of the F protein, although this mutant was not identified during a selection of MAb19 escape mutants [15].

Previous results had identified K433 as a critical residue for the binding of ch101F. Mutation of K433 to D, L, N, Q, R, or T abolished ch101F binding as previously reported [20], but, with the exception of K433D and K433R, modest to no effects upon palivizumab binding. These mutations also reduced the binding of MAb19 to various degrees highlighting the complexity of antigenic site IV/V/VI. It is interesting to note that while mutation of K433 to threonine had such a marked effect upon ch101F binding, mutation of K433 to the structurally similar serine had little to no effect. Mutation of K433 either increased fusion activity (K433R, K433N), reduced it by 50% (K433D, K433L, K433T), or had little to no effect (K433Q, K433S). Mutation K433D appeared to reduce binding of all three mAbs suggesting that this mutation was not efficiently expressed on the cell surface as the other mutations, which may account for its reduced fusion activity. Mutations T400A, C422S [21], N428D, and N428Q around the site IV/V/VI region had no effect upon mAb binding or fusion activity which show this is not a region of F protein hypersensitive to mutations.

The classical approach of selecting antibody escape mutant viruses is limited by the impact of such mutations upon viral growth fitness, and in general, provides a much more limited spectrum of residue changes. Antibody escape mutants selected with ch101F revealed only a single change in lysine residue 433 to threonine suggesting that there are additional constraints on this region of the protein that limit which amino acids changes are tolerated in this region. Furthermore, the only change identified for antibody escape mutant viruses selected with MAb19 was a single change at R429 to S. Interestingly, it appears that resistance to mAbs which map to antigenic site IV/V/VI requires more passages in vitro for selection [19]; however, the relative fitness of site II and site IV/V/VI escape mutants would need to be assessed in parallel to determine if this is true. The F protein shows a high degree of conservation both within and between subgroup A and B, as well as with bovine RSV. Given this high degree of conservation, it was somewhat surprising that the functional analysis of the fusion activity of the panel of mutations described here revealed that the fusion activity of the F protein is tolerant to multiple changes in the site II and IV/V/VI region. These changes included those identified from the selection of mAb escape mutants as well as other residue changes not identified in escape mutants. These results suggests that aspects other than fusion activity may limit the spectrum of changes tolerated within the F protein. Although the F protein is the only virion surface protein required for fusion and viral entry [25-27], the F protein is also essential for the formation of mature virion particles [26,28,29]. It is possible that the RNA sequence of this region contains some critical function, and/or that mutations in this region of the F protein impact some other unknown function(s) essential for virus growth. If these mutation affect the F protein, they would also suggest that antibodies such as MAb19 and ch101F may not neutralize virus solely by inhibition of fusion. Additional studies may provide direct information on the mechanism of action of HRSV neutralizing antibodies directed against the F protein such as ch101F.

A thorough characterization of the epitopes on the F protein for HRSV neutralizing mAbs may provide insights into the mechanisms of action by which mAbs against the HRSV F protein neutralize virus as well as a better understanding of the mechanism by which the F protein mediates cell fusion. Such studies may provide additional insights into the function of the F protein, and help in the selection and development of clinical candidates, such as ch101F, for next generation antibody-based prophylaxis and therapy for HRSV infections.

## Abbreviations

HRSV Human respiratory syncytial virus

F protein fusion protein

mAb monoclonal antibody

ch101F chimeric 101F

WT wild-type

## Competing interests

The authors CL, ND, PB, LG, RS, and AD declare that they are employees of Centocor, Inc. which provided supported for this work.

## Authors' contributions

CL generated reagents and performed the fusion assays. PB and ND performed the flow cytometry. LG conducted sitedirected mutagenesis of the HRSV F protein. AD and RS participated in the design, oversight of the conduct of the experiments, and interpretation of the results. All authors have read and approved the final manuscript.

## References

[B1] Stensballe LG, Devasundaram JK, Simoes EA (2003). Respiratory syncytial virus epidemics: the ups and downs of a seasonal virus. Pediatr Infect Dis J.

[B2] Anderson LJ, Bingham P, Hierholzer JC (1988). Neutralization of respiratory syncytial virus by individual and mixtures of F and G protein monoclonal antibodies. J Virol.

[B3] Beeler JA, van Wyke Coelingh K (1989). Neutralization epitopes of the F glycoprotein of respiratory syncytial virus: effect of mutation upon fusion function. J Virol.

[B4] Garcia-Barreno B, Palomo C, Penas C, Delgado T, Perez-Brena P, Melero JA (1989). Marked differences in the antigenic structure of human respiratory syncytial virus F and G glycoproteins. J Virol.

[B5] Trudel M, Nadon F, Seguin C, Payment P, Talbot PJ (1987). Respiratory syncytial virus fusion glycoprotein: further characterization of a major epitope involved in virus neutralization. Can J Microbiol.

[B6] Johnson S, Oliver C, Prince GA, Hemming VG, Pfarr DS, Wang SC, Dormitzer M, O'Grady J, Koenig S, Tamura JK, Woods R, Bansal G, Couchenour D, Tsao E, Hall WC, Young JF (1997). Development of a humanized monoclonal antibody (MEDI-493) with potent in vitro and in vivo activity against respiratory syncytial virus. J Infect Dis.

[B7] Feltes TF, Cabalka AK, Meissner HC, Piazza FM, Carlin DA, Top FH, Connor EM, Sondheimer HM (2003). Palivizumab prophylaxis reduces hospitalization due to respiratory syncytial virus in young children with hemodynamically significant congenital heart disease. J Pediatr.

[B8] DeVincenzo JP, Hall CB, Kimberlin DW, Sanchez PJ, Rodriguez WJ, Jantausch BA, Corey L, Kahn JS, Englund JA, Suzich JA, Palmer-Hill FJ, Branco L, Johnson S, Patel NK, Piazza FM (2004). Surveillance of clinical isolates of respiratory syncytial virus for palivizumab (Synagis)-resistant mutants. J Infect Dis.

[B9] Wu H, Pfarr DS, Johnson S, Brewah YA, Woods RM, Patel NK, White WI, Young JF, Kiener PA (2007). Development of motavizumab, an ultra-potent antibody for the prevention of respiratory syncytial virus infection in the upper and lower respiratory tract. J Mol Biol.

[B10] Young JF, Koenig S, Johnson LS (2004). Anti-RSV Antibodies.

[B11] Zhao X, Liu E, Chen FP, Sullender WM (2006). In vitro and in vivo fitness of respiratory syncytial virus monoclonal antibody escape mutants. J Virol.

[B12] Zhao X, Sullender WM (2005). In vivo selection of respiratory syncytial viruses resistant to palivizumab. J Virol.

[B13] Zhao X, Chen FP, Megaw AG, Sullender WM (2004). Variable resistance to palivizumab in cotton rats by respiratory syncytial virus mutants. J Infect Dis.

[B14] Zhao X, Chen FP, Sullender WM (2004). Respiratory syncytial virus escape mutant derived in vitro resists palivizumab prophylaxis in cotton rats. Virology.

[B15] Taylor G, Stott EJ, Furze J, Ford J, Sopp P (1992). Protective epitopes on the fusion protein of respiratory syncytial virus recognized by murine and bovine monoclonal antibodies. J Gen Virol.

[B16] Everitt DE, Davis CB, Thompson K, DiCicco R, Ilson B, Demuth SG, Herzyk DJ, Jorkasky DK (1996). The pharmacokinetics, antigenicity, and fusion-inhibition activity of RSHZ19, a humanized monoclonal antibody to respiratory syncytial virus, in healthy volunteers. J Infect Dis.

[B17] Wyde PR, Moore DK, Hepburn T, Silverman CL, Porter TG, Gross M, Taylor G, Demuth SG, Dillon SB (1995). Evaluation of the protective efficacy of reshaped human monoclonal antibody RSHZ19 against respiratory syncytial virus in cotton rats. Pediatr Res.

[B18] Arbiza J, Taylor G, Lopez JA, Furze J, Wyld S, Whyte P, Stott EJ, Wertz G, Sullender W, Trudel M (1992). Characterization of two antigenic sites recognized by neutralizing monoclonal antibodies directed against the fusion glycoprotein of human respiratory syncytial virus. J Gen Virol.

[B19] Lopez JA, Bustos R, Orvell C, Berois M, Arbiza J, Garcia-Barreno B, Melero JA (1998). Antigenic structure of human respiratory syncytial virus fusion glycoprotein. J Virol.

[B20] Wu SJ, Schmidt A, Beil EJ, Day ND, Branigan PJ, Liu C, Gutshall LL, Palomod C, Furze J, Taylor G, Melero JA, Tsui P, Del Vecchio AM, Kruszynski M Characterization of the epitope for anti human respiratory syncytial virus F protein monoclonal antibody 101F using synthetic peptides and genetic approaches. J Gen Virol.

[B21] Day ND, Branigan PJ, Liu C, Gutshall LL, Luo J, Melero JA, Sarisky RT, Del Vecchio AM (2006). Contribution of cysteine residues in the extracellular domain of the F protein of human respiratory syncytial virus to its function. Virol J.

[B22] Cardenas S, Auais A, Piedimonte G (2005). Palivizumab in the prophylaxis of respiratory syncytial virus infection. Expert Rev Anti Infect Ther.

[B23] Johnson S, Griego SD, Pfarr DS, Doyle ML, Woods R, Carlin D, Prince GA, Koenig S, Young JF, Dillon SB (1999). A direct comparison of the activities of two humanized respiratory syncytial virus monoclonal antibodies: MEDI-493 and RSHZl9. J Infect Dis.

[B24] Crowe JE, Firestone CY, Crim R, Beeler JA, Coelingh KL, Barbas CF, Burton DR, Chanock RM, Murphy BR (1998). Monoclonal antibody-resistant mutants selected with a respiratory syncytial virus-neutralizing human antibody fab fragment (Fab 19) define a unique epitope on the fusion (F) glycoprotein. Virology.

[B25] Karron RA, Wright PF, Crowe JE, Clements-Mann ML, Thompson J, Makhene M, Casey R, Murphy BR (1997). Evaluation of two live, cold-passaged, temperature-sensitive respiratory syncytial virus vaccines in chimpanzees and in human adults, infants, and children. J Infect Dis.

[B26] Teng MN, Collins PL (1998). Identification of the respiratory syncytial virus proteins required for formation and passage of helper-dependent infectious particles. J Virol.

[B27] Teng MN, Whitehead SS, Collins PL (2001). Contribution of the respiratory syncytial virus G glycoprotein and its secreted and membrane-bound forms to virus replication in vitro and in vivo. Virology.

[B28] Techaarpornkul S, Barretto N, Peeples ME (2001). Functional analysis of recombinant respiratory syncytial virus deletion mutants lacking the small hydrophobic and/or attachment glycoprotein gene. J Virol.

[B29] Techaarpornkul S, Collins PL, Peeples ME (2002). Respiratory syncytial virus with the fusion protein as its only viral glycoprotein is less dependent on cellular glycosaminoglycans for attachment than complete virus. Virology.

[B30] Branigan PJ, Liu C, Day ND, Gutshall LL, Sarisky RT, Del Vecchio AM (2005). Use of a novel cell-based fusion reporter assay to explore the host range of human respiratory syncytial virus F protein. Virol J.

